# Effects of four antiplatelet/statin combined strategies on immune and inflammatory responses in patients with acute myocardial infarction undergoing pharmacoinvasive strategy: Design and rationale of the B and T Types of Lymphocytes Evaluation in Acute Myocardial Infarction (BATTLE-AMI) study: study protocol for a randomized controlled trial

**DOI:** 10.1186/s13063-017-2361-1

**Published:** 2017-12-19

**Authors:** Francisco A. H. Fonseca, Maria Cristina Izar, Ieda M. L. Maugeri, Otavio Berwanger, Lucas P. Damiani, Ibraim M. Pinto, Gilberto Szarf, Carolina N. França, Henrique T. Bianco, Flavio T. Moreira, Adriano Caixeta, Claudia M. R. Alves, Aline Soriano Lopes, Aline Klassen, Marina F. M. Tavares, Henrique A. Fonseca, Antonio C. C. Carvalho, for the BATTLE-AMI Investigators

**Affiliations:** 1 Universidade Federal de São Paulo, Rua Loefgren 1350, 04040-001 São Paulo, SP Brazil; 2 Hospital do Coração, Rua Desembargador Eliseu Guilherme, 147, São Paulo, Brazil; 3 Instituto Dante Pazzanese de Cardiologia, Avenida Dante Pazzanese 500, São Paulo, Brazil; 4 Universidade Santo Amaro, Rua Professor Enéas de Siqueira 340, São Paulo, Brazil; 5 Universidade de São Paulo, Avenida Professor Lineu Prestes, 748, São Paulo, Brazil

**Keywords:** Acute myocardial infarction, B lymphocytes, cardiac magnetic resonance imaging, metabolomics, proteomics

## Abstract

**Background:**

Early reperfusion of the occluded coronary artery during acute myocardial infarction is considered crucial for reduction of infarcted mass and recovery of ventricular function. Effective microcirculation and the balance between protective and harmful lymphocytes may have roles in reperfusion injury and may affect final ventricular remodeling.

**Methods/design:**

BATTLE-AMI is an open-label, randomized trial comparing the effects of four therapeutic strategies (rosuvastatin/ticagrelor, rosuvastatin/clopidogrel, simvastatin plus ezetimibe/ticagrelor, or simvastatin plus ezetimibe/clopidogrel) on infarcted mass and left ventricular ejection fraction (LVEF) (blinded endpoints) in patients with ST-segment elevation myocardial infarction submitted to fibrinolytic therapy before coronary angiogram (pharmacoinvasive strategy). All patients (n = 300, 75 per arm) will be followed up for six months. The effects of treatment on subsets of B and T lymphocytes will be determined by flow-cytometry/ELISPOT and will be correlated with the infarcted mass, LVEF, and microcirculation perfusion obtained by cardiac magnetic resonance imaging. The primary hypothesis is that the combined rosuvastatin/ticagrelor therapy will be superior to other therapies (particularly for the comparison with simvastatin plus ezetimibe/clopidogrel) for the achievement of better LVEF at 30 days (primary endpoint) and smaller infarcted mass (secondary endpoint) at 30 days and six months. The trial will also evaluate the improvement in the immune/inflammatory responses mediated by B and T lymphocytes. Omics field (metabolomics and proteomics) will help to understand these responses by molecular events.

**Discussion:**

BATTLE-AMI is aimed to (1) evaluate the role of subsets of lymphocytes on microcirculation improvement and (2) show how the choice of statin/antiplatelet therapy may affect cardiac remodeling after acute myocardial infarction with ST elevation.

**Trial registration:**

ClinicalTrials.gov, NCT02428374. Registered on 28 September 2014.

**Electronic supplementary material:**

The online version of this article (doi:10.1186/s13063-017-2361-1) contains supplementary material, which is available to authorized users.

## Background

An early invasive strategy after fibrinolysis has been proven safe and effective among patients with ST-segment elevation myocardial infarction (STEMI) [1, 2]. However, during acute myocardial infarction (MI), some ligands of the injured tissue are recognized by the innate immune system, triggering the mobilization of inflammatory cells. Yet experimental studies reported that subsets of B lymphocytes may affect the healing process of injured tissue due to the mobilization of monocytes, subsequently affecting ventricular remodeling [3–6]. An increased number of circulating platelets and monocyte microparticles also seems to be related to the severity of the acute MI [7]. Furthermore, it is possible that subsets of lymphocytes and derived microparticles from B or T cells contribute additionally to myocardial injury after reperfusion, due to the release of highly inflammatory interleukins or by affecting thrombus formation [8].

### Subsets of B cells

**Fig. 1:**
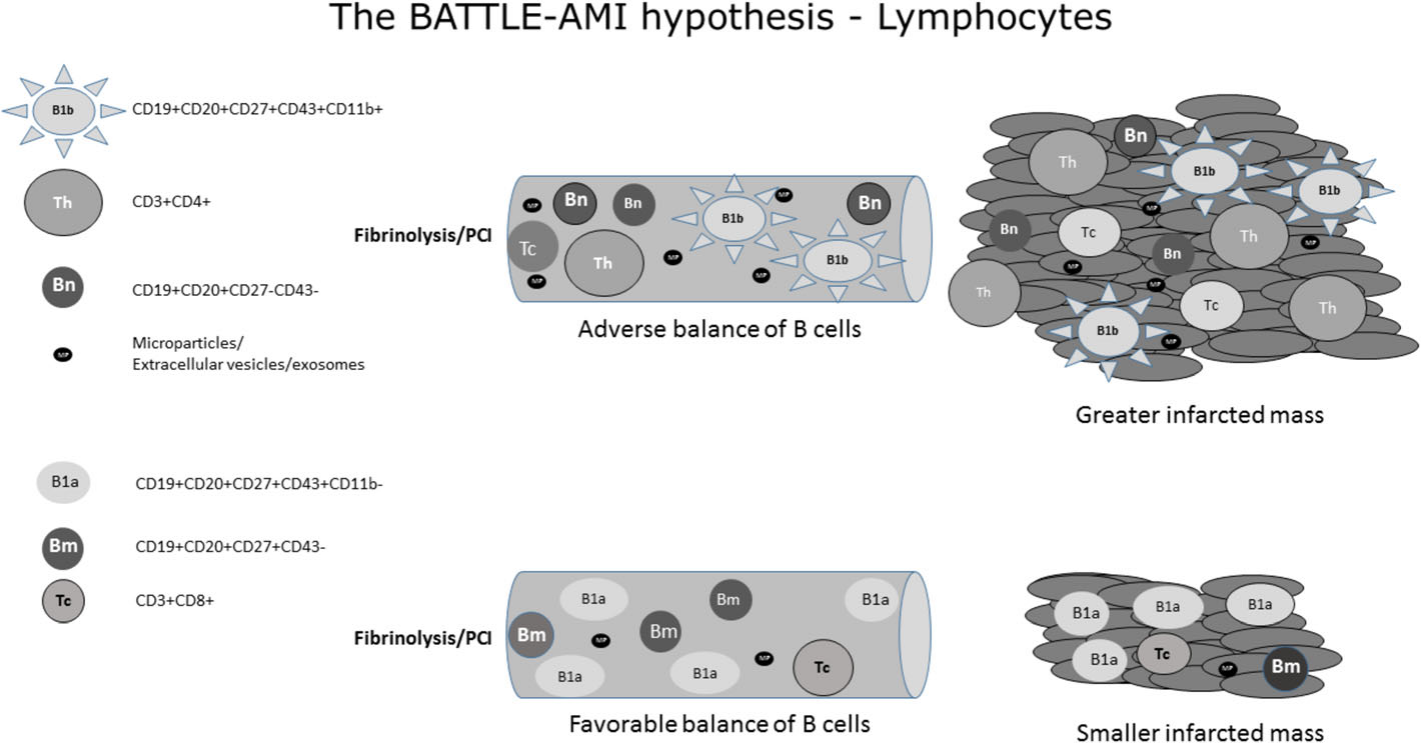
The BATTLE-AMI hypothesis – lymphocytes. After successful coronary reperfusion by a pharmacoinvasive strategy, patients with STEMI might have greater or smaller infarcted mass depending on the balance of B and T lymphocytes. B CD11b– and B memory cells seem to be related to smaller infarcted mass and better left ventricular ejection fraction (LVEF). Conversely, B CD11b + and B-naive cells are possibly related to greater infarcted mass. The role of B and T derived microparticles, extracellular vesicles, or exosomes in the ischemic tissue after coronary reperfusion is not yet established. *PCI* percutaneous coronary intervention

Experimental studies suggest an important role for B cells in atherogenesis. Initial evidence came from splenectomized ApoE^–/–^ mice developing more severe atherosclerosis compared to sham-treated mice, followed by an impressive attenuation of atherosclerosis after transfer of splenic B cells [9]; however, subsequent studies showed conflicting results and the current understanding suggests a differential role according to B cell subsets [10, 11]. The same theory might be postulated for acute MI. We hypothesized that an increased number of human B CD11b– cells may decrease infarcted mass after reperfusion of an occluded coronary artery. In addition, an increased number of B2 cells, which are considered proatherogenic, may increase the infarcted mass [12]. Other possible players such as highly inflammatory T cells, lymphocyte-derived microparticles, extracellular vesicles, and exosomes may also reach the damaged myocardium, affecting the clotting process [8], oxidative stress [13], and microcirculation [14], thus influencing the recovery of the ischemic tissue [14]. We previously reported that hyperlipidemic individuals treated with rosuvastatin presented better immune responses (higher titers of anti-oxLDL) than those receiving simvastatin/ezetimibe, thus suggesting that the choice of lipid lowering therapy may have possible beneficial role in the acute phase of myocardial infarction [15]. Figure 1 summarizes the study hypothesis regarding B and T lymphocytes.

### Microcirculation and the role of ticagrelor/rosuvastatin

In addition to its antiplatelet properties via P2Y12 receptor antagonism, ticagrelor (but not clopidogrel) increases adenosine plasma levels in individuals with acute coronary syndromes [16]. An increase in adenosine concentration following ticagrelor use seems related to the inhibition of the adenosine transporter ENT1 (type 1 equilibrative nucleoside transporter) [17]. This effect of ticagrelor increases coronary blood flow and might be important in the early protection of the ischemic tissue in the acute phase of MI [18].

**Fig. 2:**
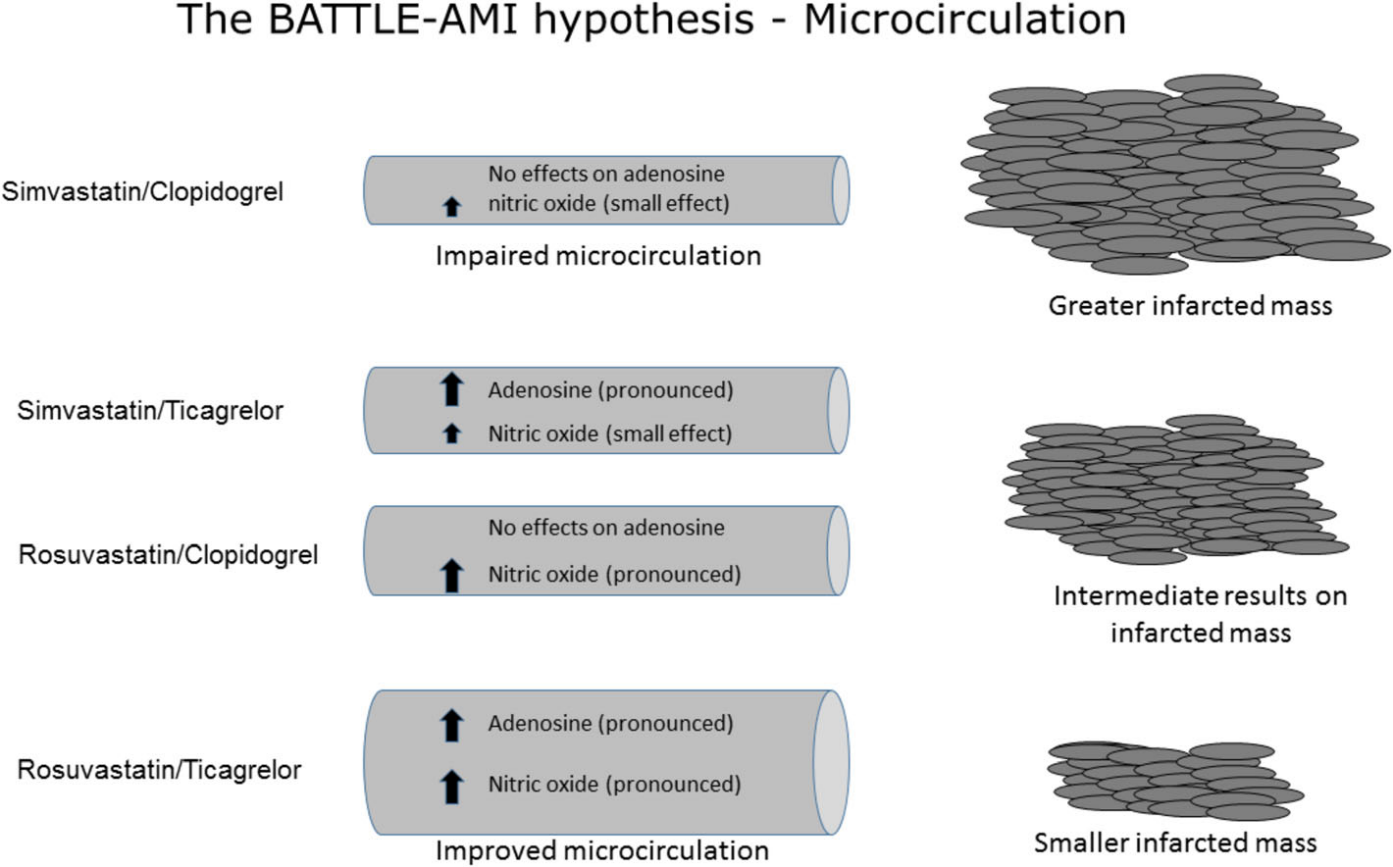
The BATTLE-AMI hypothesis – microcirculation. Microcirculation will be improved by adenosine and nitric oxide. Ticagrelor increases intra- and extracellular levels of adenosine by blocking the adenosine transporter ENT1. Rosuvastatin increases nitric oxide availability through the decrease in the intracellular signaling mediated by Rho GTPases. Decrease in Rho protein is followed by increased bioavailability of nitric oxide, promoting vasodilation. Thus, the synergism between rosuvastatin and ticagrelor will improve the microcirculation of the ischemic myocardium decreasing the final infarcted mass. The absence of effect in adenosine levels following clopidogrel use and the characteristics of simvastatin as a prodrug might have lower beneficial effect on the microcirculation

Rosuvastatin is an active substrate for hydroxymethylglutaryl-Coenzyme A reductase, decreasing the pathway of endogenous cholesterol synthesis. However, pleiotropic effects of statins seem to be mediated by the inhibition of isoprenoids, such as farnesylpyrophosphate and geranylgeranyl pyrophosphate, intermediate substances in the endogenous cholesterol synthesis, required for post-translational changes of small proteins. Consequently, there is a decrease in the intracellular signaling mediated by Rho GTPases. Decrease in Rho protein is followed by increased bioavailability of nitric oxide, promoting vasodilation [19]. Simvastatin is a prodrug that needs metabolization via cytochrome P450, isoenzymes 3A4. Only after this step, the formed metabolites serve as substrates for the hydroxymethylglutaryl CoA reductase, allowing the inhibition of cholesterol synthesis and promoting pleiotropic effects. However, thyenopyridines are also substrates for the same microsomal isoenzymes and some degree of pharmacokinetic interaction can be expected [20, 21]. Thus, another hypothesis of the BATTLE-AMI study is that by acting synergistically, the use of ticagrelor plus rosuvastatin could promote greater improvement in the microcirculation (Fig. 2). Previous studies have shown that the use of high-dose statin decreases biomarkers of myocardial damage in patients undergoing cardiac percutaneous coronary intervention, suggesting early improvement in microcirculation [22, 23].

## Methods

### Study population and randomization

The BATTLE-AMI study (NCT02428374) will include approximately 300 men and women aged > 18 years with documented STEMI submitted to fibrinolytic therapy (tenecteplase, TNK) in the first 6 h of onset of symptoms in public hospitals of São Paulo, Brazil, as part of the SP STEMI treatment network (NCT01791764) [24]. In the network routine, patients are subsequently transferred to a tertiary teaching hospital and undergo systematic early invasive coronary angiography (<24 h, designated as pharmacoinvasive strategy). After obtaining written informed consent in the teaching hospital and before coronary angiography, eligible patients will be randomized using a central computerized system in the first 24 h to one of the four open-label treatment assignments (daily doses) in a 1:1:1:1 ratio: rosuvastatin 20 mg/ticagrelor 180 mg, rosuvastatin 20 mg/clopidogrel 75 mg, simvastatin 40 mg plus ezetimibe 10 mg/ticagrelor 180 mg, or simvastatin 40 mg plus ezetimibe 10 mg/clopidogrel 75 mg. Patients with prior MI, a history of revascularization or stroke, contraindication to study drugs, chronic kidney disease, active liver disease, malignancies, hematological or rheumatic diseases, or showing clinical instability requiring rescue catheterization will be excluded. Other anti-platelet agents or lipid-lowering drugs than those assigned to participants will be prohibited.

Individuals with adverse harmful side effects related to study drugs will be discontinued from trial protocol. Monitoring of adherence to study drugs will be performed by study drug return. Assessment of adherence to study medication and concomitant treatment, as well as patient retention, will be performed at each medical visit and by phone calls. Individuals that discontinue study medication will be followed in the study center according to the scheduled visits of the study.

### Study design and objectives

**Fig. 3:**
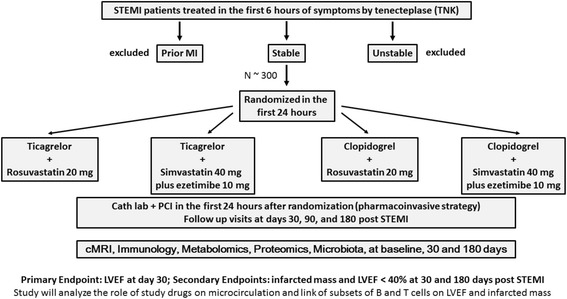
The BATTLE-AMI study *flowchart*. After fibrinolytic therapy performed in the 6 h after onset of symptoms, subjects with STEMI will be referred to the hospital for coronary angiography and PCI, if necessary. Those patients admitted to the hospital within 24 h of STEMI will be randomized to one of the four assignments in a 1:1:1:1 ratio (ticagrelor/rosuvastatin, ticagrelor/simvastatin plus ezetimibe, clopidogrel/rosuvastatin, or clopidogrel/simvastatin plus ezetimibe). Blood, urine, and feces samples will be collected at baseline, 30 days, and 180 days for metabolomics, proteomics, microbiota, and flow-cytometry studies. cMRI studies will be performed during peri-hospitalization period and at 30 and 180 days

The study design of BATTLE-AMI is shown in Fig. 3. The primary objective of the study is to compare the effects of the different antiplatelet/statin therapies in left ventricular ejection fraction (LVEF) obtained by cardiac magnetic resonance imaging (cMRI) 30 days post STEMI. Secondary objectives include the comparison of treatments on infarcted mass and the percentage of participants with LVEF < 40% (by cMRI) at 30 and 180 days of follow-up. In addition, the relation of subsets of B and T lymphocytes with the infarcted mass and LVEF will be examined in samples collected in the first 24 h and at 30 and 180 days post STEMI. Analyses of microbiota, metabolomics, and proteomics will be performed at the same time points aiming at evaluating the lymphocyte differentiation and metabolites related to cMRI parameters. Endothelial progenitor cells and microparticles derived from platelets, endothelium, monocytes, and lymphocytes will be quantified at the same time points and their relationship with cMRI parameters will be evaluated.

Our main hypothesis is that rosuvastatin/ticagrelor will be superior than simvastatin plus ezetimibe/clopidogrel, for the cMR parameters. Additionally, the pilot study indicated that the ezetimibe/clopidogrel was similar to simvastatin plus ezetimibe/ticagrelor and rosuvastatin/clopidogrel combinations regarding the primary endpoint and we decided to compare those other groups (rosuvastatin/clopidogrel and simvastatin plus ezetimibe/ticagrelor) to explore possible mechanisms for the primary and secondary objectives.

Thereafter, our sample size derivation was done considering that rosuvastatin/ticagrelor will improve mean cMRI LVEF by 6% compared to each of the other three groups, assuming a 13% dropout rate. The alpha level fixed in 0.05/3 (1.67%) was considered to adjust the global alpha error in 5% using Dunnett’s test approach (many-to-one comparisons).

### Study organization

The steering committee of the study is composed of representatives from the Universidade Federal de São Paulo (UNIFESP, São Paulo, Brazil), Instituto Dante Pazzanese de Cardiologia (IDPC, São Paulo, Brazil), and Research Institute, Hospital do Coração (HCor, São Paulo, Brazil). An independent data safety monitoring board was constituted by representatives of the Heart Institute (InCor, São Paulo, Brazil) and is planned to analyze data three times during the study to evaluate only safety measures (serious adverse events, such as bleeding, acute kidney injury, new myocardial infarction, and mortality). Based on those parameters, the board may consider removing an intervention arm or study’s early termination by safety reasons or LVEF data. Those comparisons will consider Haybittle-Peto boundary (*p* < 0.001).

### Laboratory analyses

Microparticles and endothelial progenitor cells will be determined by flow-cytometry using specific markers [25–27]. Subsets of B and T lymphocytes will be evaluated by flow-cytometry and IgM and interleukins released by specific B and T cells will be quantified by ELISA for cytokine evaluation and ELISPOT for IgM detection [28]. Metabolomics and proteomics in plasma and urine will be determined by LC-MS/MS or CE-MS technique [29]. A methodology for targeted and untargeted metabolomics will be evaluated by using a pool of bio fluids (urine and plasma) with some (*n* = 10) healthy people. All samples will be submitted to a protein precipitation procedure, to guarantee method repeatability and preserve the chromatographic column. After protein precipitation, the supernatant will be dried followed by addition of a proper solvent and analyzed by LC-MS/MS or CE-MS. For untargeted metabolomics, in order to evaluate possible sources of variation during analysis, Quality control samples (QC) will be processed, which consist of a pool of 5 μL of each sample (control and treated). Thus, the QCs will be injected five times in the beginning of the batch, between every five injections and three at the end of the batch, and for the targeted metabolomics method, standards and internal standards will be used.

All data obtained for untargeted metabolomics will be processed by specific software and all significant metabolites will be obtained after statistical analysis by SIMCA 14 software (Umetrics, Umea, Sweden) by multivariate analysis. All putative metabolites will be analyzed by using the HMDB and METLIN free databases followed by KEGG pathway evaluation. Otherwise, the protein pellet obtained will be dissolved and the proteins digested according to a well-established procedure [29, 30]. For proteomics, all peptides will be detected by LC-MS/MS and identified using specific programs to proteomics field. The proteins differentially expressed in the four groups, including the three visits, will be compared.

Microbiota will be analyzed by new-generation sequencing techniques followed by bioinformatics analyses [31].

### Cardiac magnetic resonance images

The amount of infarcted mass, LVEF, and microcirculation will be determined by 3-T cMRI. For left ventricular function, cMRI images will be acquired using a 3-T scanner. Patients will be positioned in the supine position with a phased-array coil placed over the thorax. Repeated breath-holds and gating to the electrocardiogram will be applied to minimize the influence of cardiac and respiratory motion on data collection. Cine cMRI will be performed using a steady-state free-precession technique (fast imaging employing steady-state acquisition). We will obtain cine images in the two-chamber, four-chamber, left ventricular outflow tract, and short-axis views, the later with the first slice positioned at the basis covering the mitral valve and the last slice covering the apex, resulting in 10–12 cine breath-hold short-axis images to cover the entire left ventricle. Ischemia detection will be performed using first-pass perfusion imaging in the short-axis orientation only, with at least three slices (the maximum number of slices will be limited by heart rate). Infarction detection and quantification images will be acquired using the myocardial delayed enhancement technique, after the injection of a commercially available gadolinium-based contrast agent, administered intravenously at a dose of 0.15 mmol per kilogram of body weight. Contrast-enhanced images will be acquired in the same views as those used for cine MRI, with the use of a segmented inversion-recovery sequence. Each patient study will be reviewed by two independent blinded readers using dedicated software. LV function will be calculated using cine images to measure LVEF, volumes, and mass according to standard methods. Perfusion defects will be determined solely by subjective visualization. Perfusion defects will be defined as focal regions of myocardium that had diminished and/or delayed contrast enhancement compared with normal myocardium. For each patient study, each reader will report the likelihood of myocardial ischemia on a scale of 1 to 3: 1 = definitely normal; 2 = possibly abnormal; and 3 = definitely abnormal. Delayed-enhancement images will be used for infarct characterization. Myocardial tissue for each patient will be classified as hyper-enhanced (scar tissue) or normally enhanced myocardium after the observer, through manual interaction, defined a region of interest (ROI) within remote non-infarcted territory. The endocardial and epicardial borders will also be defined by manual interaction. Hyper-enhanced tissue is defined as areas with a signal intensity of > 2 standard deviations from the mean signal intensity measured in remote areas within the manually predefined ROI.

### Angiographic analysis

Patients will undergo coronary angiography in the first 24 h of STEMI and percutaneous intervention when needed. An independent angiographic laboratory will assess all angiograms for baseline and post-PCI lesion quantitative coronary angiography (Medis Medical Imaging System, Leiden, The Netherlands), blinded to randomization assignment and clinical outcomes (Escola Paulista de Medicina, Unifesp, São Paulo). TIMI flow in the infarct vessel will be assessed as previously reported [32], in which blood flow with a grade of < 3 (0–2) indicates suboptimal flow and blood flow with a grade of 3 indicates normal flow within the vessel. Myocardial blush grade and TIMI frame count will be used to quantify myocardial tissue level perfusion. Quantitative coronary analysis will include both baseline and post procedure: proximal, distal and interpolated reference diameter; percent stenosis; minimal lumen diameter; and lesion length. Qualitative analysis will include: the presence of thrombus; tortuosity; calcification; plaque rupture; and aneurisms. The Syntax score (SS) will be calculated by an experienced interventional cardiologist blinded to treatment assignment, type of stent used and clinical outcomes. Each lesion with ≥ 50% diameter stenosis in vessels ≥ 1.5 mm will be scored using the SS algorithm, which has been fully described elsewhere [33, 34] and is available on the SS website (www.syntaxscore.com).

### Data collection and analyses

**Fig. 4:**
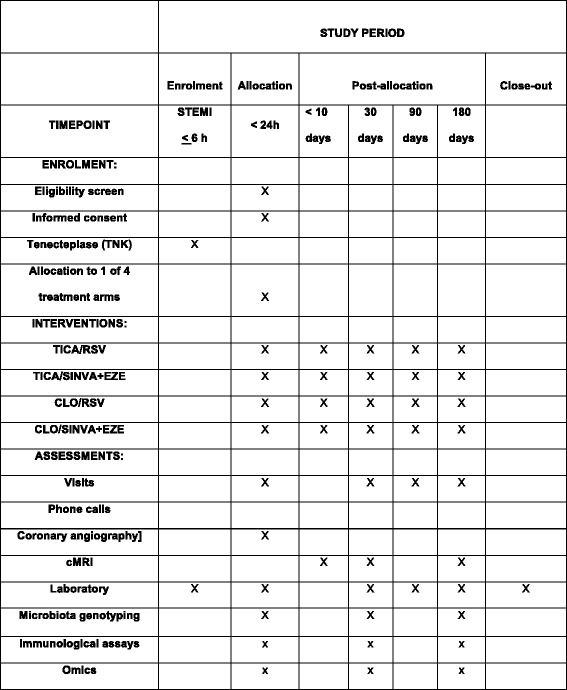
Schedule of enrolment, interventions, and assessments. *TICA* ticagrelor, *RSV* rosuvastatin, *Sinva* simvastatin, *CLO* clopidogrel, *EZE* ezetimibe, *cMRI* cardiac magnetic resonance imaging

Data collection will be performed centrally. Recommendations for Interventional Trials (SPIRIT) is described in Fig. 4. A SPIRIT Checklist is included as an Additional file (Additional file 1).

This is an open label study, with outcome assessors and data analysts blinded to treatment assignment.

## Discussion

The BATTLE-AMI study will address important topics in the treatment of STEMI after a pharmacoinvasive strategy. First, the trial will examine whether the use of rosuvastatin/ticagrelor will be followed by improvement in the LVEF and smaller infarcted mass. The mechanisms involved will be examined by metabolomics and proteomics, including the measurement of adenosine. Second, the trial will evaluate the role of subsets of lymphocytes in the final infarcted mass and ventricular remodeling, exploring B and T cells phenotypes. For this analysis, the absolute number of subtypes of lymphocytes will be correlated with cMRI parameters as well as with those parameters obtained in the coronary angiogram. We expect that increased number of lymphocytes of more inflammatory phenotypes will be related to reperfusion injury, thus influencing the final infarcted mass and LVEF at 30 days post MI. Third, the effects of microbiota on the cMRI parameters and lymphocytes differentiation will be examined. We expect that the presence of a pathological microbiota may contribute to lymphocyte phenotype differentiation. Finally, the relationship among microparticles derived from endothelium, platelets, and leukocytes with the cMRI parameters will be addressed. This analysis will test the relevance of these new vascular biomarkers in the coronary heart disease and possible influences of the treatments. Thus, the trial will review the role of two major players considered to be involved with ventricular remodeling and infarcted mass size: inflammation and microcirculation under highly effective lipid lowering therapies.

### Trial status

The first patient was included in the trial on May 2015 and patient recruitment is ongoing.

## Additional file

Additional file 1: SPIRIT checklist. (DOC 122 kb)

## Abbreviations

CE-MS: Capillary electrophoresis-mass spectrometry
cMRI: Cardiac magnetic resonance imaging
ELISA: Enzyme-linked immunosorbent assay
ELISPOT: Enzyme-linked ImmunoSpot
ENT1: Type 1 equilibrative nucleoside transporter
HMDB: Human Metabolome Database
KEGG: Kyoto Encyclopedia of Genes and Genomes
LC-MS/MS: Combination of liquid chromatography with mass spectrometry
LVEF: Left ventricular ejection fraction
METLIN: Metabolomics database
MI: Myocardial infarction
RhoGTPases: Enzymes of Rho family that hydrolyze guanosine triphosphate
STEMI: ST-segment elevation myocardial infarction
TIMI flow: Thrombolysis in myocardial infarction score system for levels of coronary blood flow
